# Greater than the sum of our parts: The new division of labor in design with AI

**DOI:** 10.1093/pnasnexus/pgag208

**Published:** 2026-06-22

**Authors:** Jessica Menold, Christopher McComb, Conrad Tucker, Panos Papalambros

**Affiliations:** Mechanical and Industrial Engineering, The Pennsylvania State University, University Park, PA 16802, USA; Mechanical Engineering, Carnegie Mellon University, Pittsburgh, PA 15213, USA; Mechanical Engineering, Carnegie Mellon University, Pittsburgh, PA 15213, USA; Mechanical Engineering, University of Michigan, Ann Arbor, MI 48109, USA

**Keywords:** AI, digital twins, design, design science, automation

## Abstract

Design is how humans change the world, and in the age of AI it is increasingly a joint activity between humans and machines. This perspective argues that AI does not simply add new tools to the designer’s repertoire; it has the capability to reorganize the division of labor in design and, in doing so, reshape what it means to be a designer. We distinguish between *representative* technologies, which model and derisk complex systems (eg digital twins, immersive simulations), and *operative* technologies, which act directly within design processes (eg generative and agentic systems that propose, evaluate, and select alternatives). Viewed through the Five-Cycle model of design, we argue that these technologies widen inputs, accelerate exploration, and tighten feedback across problem definition, conceptual and embodiment design, and value proposition. In our model, representative tools derisk what to believe; operative tools derisk what to try. Together these technologies could enable closed-loop, hybrid human-AI design processes in which human roles shift from manual problem solving toward stewardship, curation, translation, and, in a democratized future, even historical guardianship of designerly knowledge. We contend that design science is essential for understanding and guiding this transition: explaining how human purpose, creativity, and responsibility are redistributed in human-AI teams; developing methods to study how values propagate through automated design workflows; and informing education and practice so that increasingly automated design processes remain aligned with human intent and societal well-being.

## The changing landscape of engineering design

We situate our examination of advanced technologies within engineering design, informed by the authors’ experience in design research, education, and practice. Over the past century, computation has progressively expanded designers’ ability to represent, analyze, and iterate, from early engineering analyses to computer aided desgin or computer aided engineering (CAD/CAE) and simulation. With today’s rapid advances in AI and supporting digital infrastructure, the central question is shifting from how tools support designers to how human and machine roles are being reorganized within the design process.

**Figure 1:**
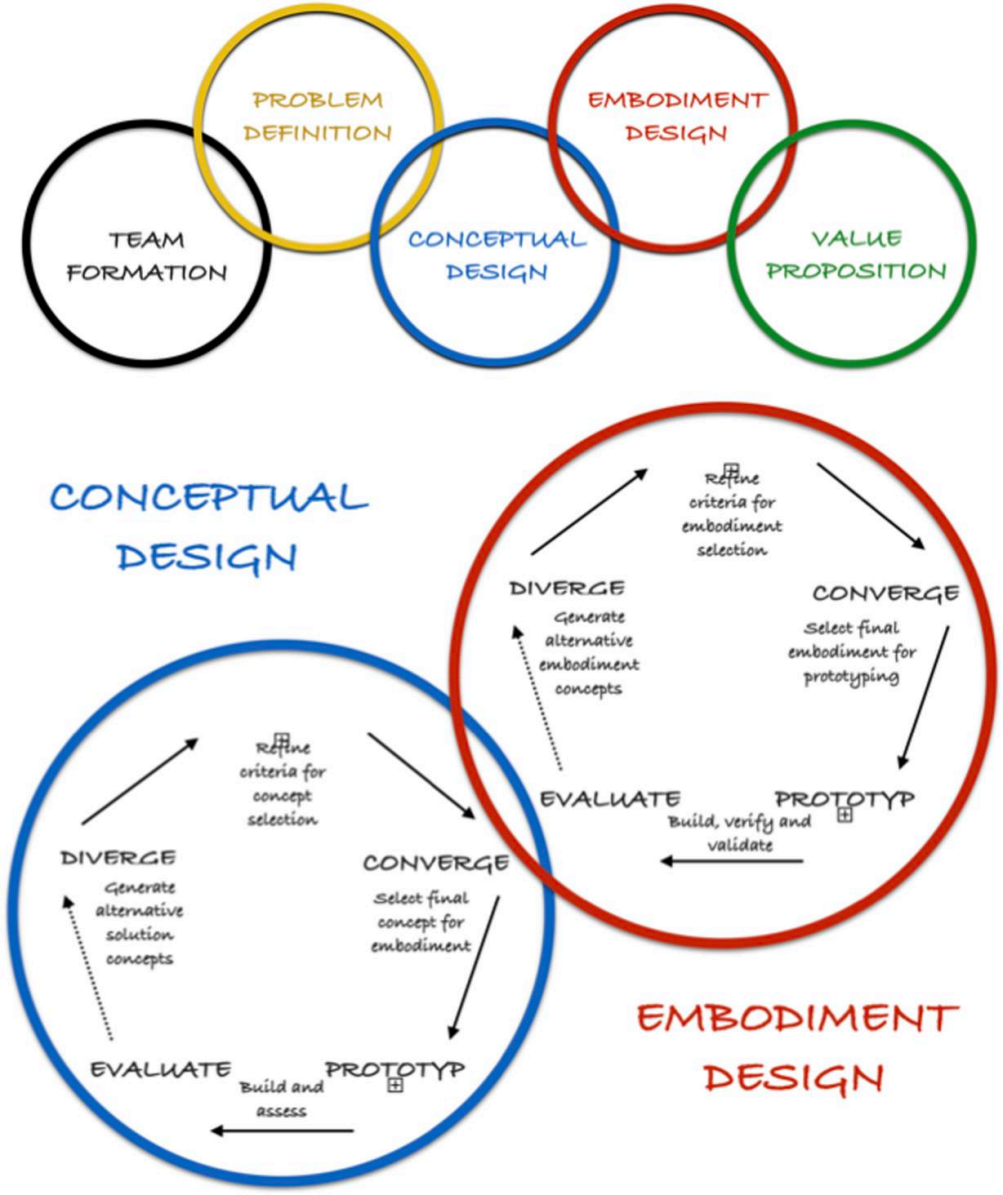
Top: the 5C model of the design process; bottom: structure of activities inside the conceptual and embodiment design phases.

To ground the discussion, we use the Five-Cycle (5C) model of design (Fig. 1) (1). The cycles describe recurring activities in team formation, problem definition, conceptual design, embodiment design, and value proposition. Together they provide a simple scaffold for locating where technologies intervene, specifically, what inputs they widen, what activities they accelerate, and what feedback they tighten across phases.

The 5C model structures our perspective by locating where and how emerging technologies intervene in design. Many contemporary challenges, such as climate change, healthcare delivery, and resilient infrastructure, are “wicked problems” (2): inherently ambiguous, value-laden, and resistant to purely technical solutions (3). Rather than treating design as detached optimization, the 5C model foregrounds subjective and systemic considerations, particularly in the problem definition and value proposition phases. This framing provides a useful scaffold for identifying which aspects of design are most susceptible—or resistant—to automation. Within this framework, representative and operative technologies play distinct but complementary roles. Representative technologies primarily augment problem definition, embodiment, and verification by structuring evidence, reducing epistemic uncertainty, and enabling earlier validation. Operative technologies, by contrast, intervene in conceptual and embodiment design by expanding the rate and breadth of exploration through automated generation, evaluation, and selection (4). Across all phases, human designers remain responsible for framing objectives, adjudicating trade-offs, and determining when computational outputs are sufficiently trustworthy to act upon. Table 1 maps these roles to the core challenges of each 5C phase.

**Table 1:** Illustrative roles of operative and representative technologies across phases of the 5C design process.

5C phase	Design challenges	Role of technology	Resultant value
C1			
Team Formation	Difficulty following roles and norms; confusing jargons and turf protection in “multifunctional” teams; incomplete knowledge representation; no follow-through across design phases	AI agents as teammates	Fill knowledge gaps in team; presumed lack of egos; consistency and quick familiarity with terminology
C2			
Problem Definition	Large and heterogeneous stakeholder inputs; ambiguous and conflicting requirements	Natural language processing, ontologies, and knowledge graphs structure requirements; detect gaps, duplications, and inconsistencies; preserve traceability to evidence	Reduces framing risk by improving what designers know and revealing what may be overlooked
C3			
Conceptual Design	Vast, under-constrained solution spaces; limited human capacity for exploration	Operative AI (generative and multiagent models) rapidly proposes and evaluates large sets of feasible concepts under explicit constraints	Changes the economics of exploration by accelerating divergence without replacing human creativity
C4			
Embodiment Design	Balancing performance, perceptual design attributes (5), manufacturability, and compliance under tighter constraints	Representative tools (digital twins, simulations) bound feasible regions; operative optimizers search within those bounds	Enables earlier testing and validation, reducing downstream rework and certification risk
C5			
Value Proposition	Comparing alternatives across competing objectives and stakeholder values, including the designer’s own values	Multicriteria decision models and synthetic user simulations support comparative evaluation	Makes value trade-offs explicit across cost, sustainability, safety, and user experience
Across All C’s	Normative judgment, ethical trade-offs, and contested values	Human oversight and intervention remain essential; AI provides evidence but not resolution	Prevents over-automation of value-laden decisions and preserves accountability

In this perspective, we review the implications of *representative technologies*, technologies designed to represent systems in silico (eg digital twins (DT) and immersive simulations), and *operative technologies*, technologies that act within design processes, such as generative agents that propose, evaluate, and select alternatives. We argue these classes function as complementary derisking mechanisms: representative tools improve what evidence is credible, while operative tools expand what actions are feasible at low cost. We illustrate this distinction with aerospace (representative-dominant) and consumer product innovation (operative-dominant), and discuss implications for research, education, and practice.

Taken together, representative and operative technologies sit atop the same substrate: abundant, instrumented data and scalable computation, increasingly enabling tightly coupled, data-driven AI enabled engineering design workflows across domains (6). Viewed through the 5C lens, this substrate widens inputs and feedback in every cycle, shaping what teams know, how they explore, how they embody concepts, and how they validate claims. These shifts translate into concrete implications for practice: broader access to disciplinary and user knowledge; expanded generation of conceptual and embodiment alternatives; and earlier validation of both objective and subjective performance, including through synthetic users.

In what follows, we trace how computation has transformed the theory and practice of design, marking the evolution from engineering design toward design science. We then examine representative and operative technologies in greater detail before concluding with implications for research, education, and practice in an age of automation.

## Design science and the evolution of design with computation

Designers’ responsibility has been recognized for millennia: societies have long held makers accountable for harm caused by failures in the built world (7). Today, that responsibility extends to complex socio-technical systems, such as, global infrastructures, autonomous technologies, and algorithmic decision systems, where consequences can be wide-reaching and difficult to anticipate. Computational representations, simulations, and preference models have expanded designers’ ability to support verification and validation, and AI further accelerates these capabilities. As tools grow more powerful, so too does the need to study design not only as technical execution but as judgment, intent, and value-laden reasoning, bridging engineering design and design science.

So far, the discussion in this perspective has been largely about design from an engineering perspective. It is a relatively small step to consider design as a scientific discipline (8–12). The proximity of design to art and the holistic view of design as an integrating activity across diverse disciplines with its own special intuition argues about the uniqueness of design thinking and “designerly” ways of knowing (13, 14). Design is also viewed as a purposeful cognitive process (15) with work within neurocognition looking at the links between design problem solving and brain activity (16, 17).

Our introductory definition of design as how humans change the world is consistent with Simon’s framing of design as the systematic process of devising courses of action to change existing situations into preferred ones (18). This vision was a pivotal one: design was recognized as an object of scientific inquiry rather than merely professional practice. Simon’s framing laid the foundation for a growing research community that generates knowledge about design and designing and propagates it through design education and practice. Design is both an empirical phenomenon and a social process (19), a discipline that creates the world as much as it studies it. Design science is both descriptive, seeking to explain how design occurs, and normative, seeking to prescribe how it ought to occur. The increasing integration of computation into design amplifies the duality of both design and design science. As digital technologies have become central to how designers think and act, they have also become vessels of embedded logic and value.

Each generation of technology embeds assumptions about what counts as a “good design,” transforming tools into carriers of normative judgment. For instance, traditional CAD systems were built around the logic of subtractive manufacturing, encoding rules and constraints that privileged machinable geometries (20). When additive manufacturing emerged, these same assumptions became limiting; CAD software simply could not represent the complex, organic forms that new fabrication methods made possible (21). This example underscores a broader shift: each new generation of computational tools extends human capability while quietly redefining what counts as possible or desirable in design, embedding normative assumptions directly within algorithms.

From this vantage point, the evolution of design with computation is not merely a story of automation or efficiency gains, but a continuous renegotiation of human and machine agency. As computational power has grown, from the era of minicomputers and CAD in the 1980s to today’s high-performance cloud computing and AI-driven optimization, so too have the fidelity, scope, and speed of design processes. Each technological leap has extended human capability, expanding what can be modeled, simulated, and optimized. Yet these same advances introduce qualitatively new questions about authorship, accountability, purpose, and values in design. As AI takes on increasingly operative roles within engineering design, the field faces a critical inflection point: how to sustain its identity as a science of the artificial while ensuring that human judgment, intention, and societal values remain at its core.

## Representative and operative technologies in design

Here, we distinguish between representative and operative technologies as two types of design technologies that are broadly applicable.

### Representative technologies

A growing body of design research and practice has begun to map how representative technologies are transforming the design process itself. These technologies are defined here as representing systems, products, and services in silico, such as DT (DT), immersive reality, knowledge graphs, ontologies and simulations. In a systematic literature review, Kreuzer et al. (22) highlight that AI-enabled DTs can be used to simulate system or product behavior, anticipate failure, and reduce risk in early stage conceptual design. For example, Mahan and Menold (23) created a DT of additive manufacturing processes and used the DT to simulate translation of engineering CAD models through a digital workflow, highlighting the errors that can be “stacked up” across stages of the design process, resulting in end parts that are significantly out of tolerance. Haynes et al. (24) further note that DTs are particularly well suited for re-design tasks, since a DT can be generated based on data from the existing system. Practically, DTs therefore can help designers visualize how early stage design decisions may ripple outward causing issues in downstream design processes or with final product specifications. Building on this work, Ferrari and Willcox (25) emphasize that DTs in mechanical and aerospace engineering have demonstrated tangible value by accelerating design cycles, reducing risk, and lowering sustainment costs through predictive analytics and continuous data integration. Similarly, Anwer et al. (26) argue that the convergence of DTs with high-fidelity simulations and AI-driven optimization represents a dramatic change in engineering design, from static modeling to dynamic, data-informed representations that extend throughout a product’s lifecycle. Together, these studies underscore how DTs enable a tighter coupling between physical and virtual domains, fostering a closed-loop design ecosystem, where insights from operation and maintenance directly inform new design iterations.

**Figure 2:**
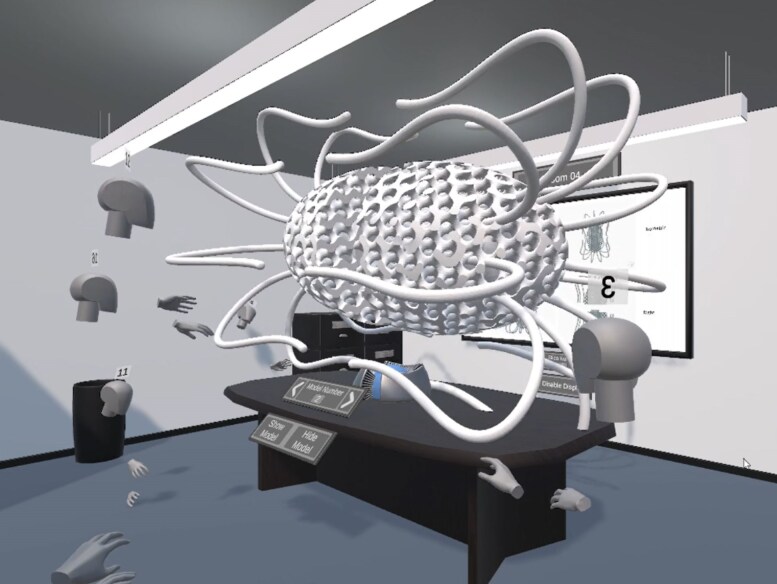
Example of VR-enabled design team working through concept generation and selection.

Complementary research has begun to examine how immersive and mixed-reality environments contribute to this representational shift by enhancing the designers’ ability to visualize, manipulate, and communicate with complex system models in real time. For instance, Pirker et al. (27) found that fully immersive virtual reality combined with DT models enables richer interaction, shared understanding, and collaborative decision-making across distributed design teams. Such environments can improve shared understanding and coordination by reducing ambiguity in spatial reasoning and situational awareness (28–31). In Fig. 2, we show an example from our own research of a virtual reality enabled design team, where individuals working remotely were able to co-locate virtually in a VR meeting room and discuss design concepts as a group. Further, in Ref. (32), we show that virtual design teams rely on high fidelity visualizations and CAD models to cross disciplinary boundaries. Critically, we also found in prior work (33) the often designers’ perception of design processes and outcomes can be biased, causing dissonance between designers’ perceptions and reality. High fidelity visualizations enabled by DT stand poised to radically reduce this dissonance, particularly for virtual teams. For example, several empirical studies have demonstrated that immersive virtual environments facilitate higher levels of engagement and coordination among team members by reducing ambiguity in spatial reasoning and improving situational awareness during complex design tasks (28–31). Our own prior work highlights that high-fidelity behavioral digital simulations of real world tasks can elicit authentic behavior from humans (34), underscoring the ability of representative technologies to augment both design practice and design research. Research on immersive visualization also emphasizes that virtual and mixed-reality tools allow users to grasp layered system behaviors and spatial relationships more intuitively than traditional 2D screens (35). However, this literature also highlights persistent challenges, such as data interoperability, model fidelity, and the cognitive trust designers place in simulated environments, as active areas of investigation requiring deeper empirical work and methodological frameworks (36).

Beyond physics-based DT and immersive simulation, a complementary class of representative technologies focuses on representing *design knowledge, intent, and context*, such as requirements, functions, rationales, constraints, stakeholders, and assumptions, all in a structured, machine-interpretable form. Ontologies and knowledge graphs can serve as representations of the design process by making relationships among needs, requirements, functions, and artifacts explicit, queryable, and auditable (37). For example, ontology-based templates and knowledge bases have been used to formalize decision logic and retrieve design rationale via structured queries, enabling traceable connections between early problem framing choices and downstream design outcomes (38). More recently, multimodal knowledge graphs have emerged as a powerful class of representative technologies, enabling designers to codify and navigate heterogeneous design data by jointly representing text, images, CAD models, simulations, and lifecycle information within a unified semantic structure (39). By supporting cross-modal linking, querying, and reasoning, these representations enhance sensemaking in early problem framing and concept exploration. Importantly, these technologies can help designers more effectively identify dissonance or inconsistencies in requirement sets across large multilevel hierarchical systems projects (37). For example, Hosseini et al. (40) demonstrated that pairing knowledge graphs with large language models (LLMs) can help designers integrate traditionally difficult and complex downstream requirements, reducing overall project costs by identifying gaps in requirement sets early on. Graph-based formalisms have also long supported computational design synthesis through graph transformation approaches (41). More recently, these formalisms are being extended through the integration of shape-grammar style operators (42) with LLMs, which can assist designers in authoring, interpreting, and operationalizing formal grammars expressed in symbolic or natural language forms (43). In this configuration, LLMs do not replace the rigor of grammars, but rather lower the barrier to specifying and manipulating them, enabling systematic exploration of families of designs while preserving formal structure and intent. Viewed through the 5C design process, knowledge representations such as ontologies and design knowledge graphs primarily strengthen the *problem definition* and *value proposition* phases. These representative technologies enable early identification of gaps, redundancies, and conflicts within large, multilevel requirement hierarchies, preserve traceable links from downstream design decisions back to upstream assumptions and evidence, and support reflection on how proposed solutions align with stakeholder values. In this way, ontological and graph-based representations increase the fidelity of designerly knowledge itself, strengthening verification, validation, and accountability throughout the design process.

Taken together, these studies suggest that representative AI technologies go beyond extending modeling capacity to fundamentally reshape the epistemic foundations of design practice by altering what designers can see, test, and trust. By making previously inaccessible aspects of systems visible, for example through digital replicas, immersive visualization, or predictive models, these technologies influence how problems are framed, trade-offs are evaluated, and decisions are made under uncertainty. Importantly, the effectiveness of representative technologies depends not on achieving uniformly “high” fidelity, but on the alignment between the form of fidelity provided and the design task at hand. Fidelity may pertain to different dimensions, including functional behavior, geometric form, data completeness, or the richness of human interaction and human perception, and representative tools often achieve high fidelity in some dimensions while remaining coarse in others. What matters, from a design science perspective, is whether the available fidelity is sufficient to support the cognitive and decision-making needs of designers at a given phase of the process, serving to extend design cognition by making critical relationships inspectable, comparable, and testable.

This dynamic is particularly evident in domains such as aerospace design, where extreme system complexity, high costs of physical prototyping, stringent verification requirements, and regulatory emphasis on safety make virtual exploration not merely advantageous but essential.

#### Case study: the role of representative technologies in aerospace design tasks

In the aerospace domain, the imperatives of safety, certification, and extreme system complexity create strong incentives for design teams to derisk early-phase work. Representative AI technologies, particularly DTs and high-fidelity simulation environments, are emerging as powerful mechanisms for doing so. Ferrari et al. (25) demonstrate that DTs in mechanical and aerospace systems deliver value by accelerating development while simultaneously reducing risk, lowering overall project costs, and mitigating the chronic issue of budget overruns that has historically plagued large aerospace programs (44). These findings align with industry-level reports showing that aerospace manufacturers increasingly treat DTs as living models that integrate design, analysis, and operations across the artifact lifecycle. When embedded early in the problem definition and conceptual design phases, DTs enable shorter design cycles, faster iteration, and more reliable product launches (45). Moenck et al. (46) note that while full system-level DTs remain aspirational, subsystem- and process-level implementations are already yielding measurable risk-reduction benefits in production and maintenance.

Despite these advances, several barriers limit the broader application of representative AI technologies in aerospace design. Chief among them are data availability and model fidelity. Systematic reviews consistently observe that most aerospace DTs are confined to later lifecycle stages, where abundant operational data exist, and that their use in conceptual design remains rare. Beyond data and fidelity, organizational and certification constraints present additional challenges. Aerospace firms face entrenched regulatory, supply-chain, and cultural barriers that make it difficult to shift verification risk from physical prototyping to virtual environments. Thus, while DTs can reduce verification risk, they cannot eliminate it. The Aerospace Industries Association and the American Institute of Aeronautics and Astronautics (45) emphasize that to realize their full potential, DTs must be integrated into conceptual design and planning rather than retrofitted after embodiment design. Finally, integration, interoperability, and standardization remain persistent obstacles. The recent report published by the Digital Twin Consortium (47), a global consortium of industry and academia advancing DT standards and best practices, highlights the lack of data-exchange standards, fragmented digital workflows, and the difficulty of aligning models and processes across organizational boundaries as major inhibitors to scaling DT use in the problem definition and conceptual design phases within aerospace industries.

From a design science perspective, these developments underscore that representative technologies do not simply accelerate existing activities within design phases, but can modify or eliminate them altogether, reshaping the design process itself. They reconfigure how and when decisions are made, by whom, and at what stage, shifting traditional boundaries of responsibility and risk. For example, when a DT enables engineers to simulate certification loads or fatigue life virtually, the decision to build a physical prototype shifts from a single milestone to an iterative decision point, where cycles of virtual testing and simulation determine when and whether physical validation is necessary. Increasingly, these representative environments are coupled with operative systems, such as optimization agents and generative design tools, that act directly on DT representations to propose design modifications that satisfy certification constraints while improving performance. In this configuration, the DT bounds the feasible solution space, while operative systems explore within those bounds, forming a closed-loop in which simulation-informed exploration and model refinement accelerate early-stage decision-making. Empirical work on aero-engine DT supports this shift: higher-fidelity models have been shown to enable earlier trade-off decisions in engine assembly design, illustrating how design accountability and timing are being fundamentally restructured (48).

### Operative technologies

While representative technologies primarily aim at the functionality of the designed artifact, operative technologies aim at the functionality of the design process itself. Specifically, we define *operative technologies* as those that generate, select, or orchestrate design decisions and downstream actions (eg generative models that produce concepts or CAD (49), multiagent systems that search a design space (50), tool-using agents that call solvers and databases (51), and workflow policies that allocate tasks across humans and machines (4)). Representative approaches primarily increase the fidelity of design verification, while operative approaches increase the bandwidth of design activities. This can fundamentally shift the economics of these activities. Human designers must intelligently choose where to allocate their effort within the design process and where to use machine effort. These choices will define the most efficient and effective designers of the future, who will need to be keenly aware of how they uniquely add value.

**Figure 3:**
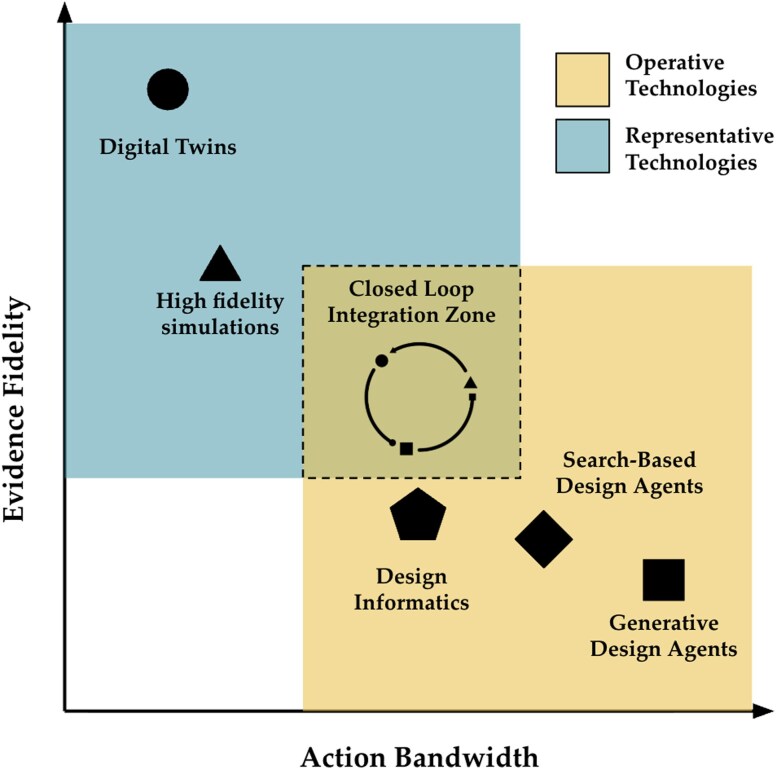
Representative tools raise evidence fidelity; operative tools expand action bandwidth; together they form a closed-loop for derisking and exploration.

In recent discourse, *generative design agents* have become the most visible face of operative AI in design. These make use of learned generators and tool-using /////LLM workflows to rapidly propose concepts, sketches, specifications, and even CAD-like representations. However, operative technologies extend beyond generative models alone. A longstanding class of *search-based design agents* produces alternatives by navigating a design space through iterative proposal and selection—using stochastic operators (eg evolutionary algorithms, simulated annealing, or move-based policies) coupled to explicit objectives, constraints, and evaluators (50). Rather than synthesizing artifacts directly, these systems instantiate a policy for where to search next and when to accept or reject candidate moves. Ultimately, these differing approaches provide complementary strengths with respect to action bandwidth and evidence fidelity (see Fig.3).

Operative technologies go beyond generative models and search-based systems, and include systems that act on design knowledge to advance work products, which we refer to broadly as *design informatics*. This may include, for example, leveraging LLMs paired with ontologies and knowledge graphs to generate contextually relevant design actions or recommendations (52). Retrieval-augmented generation (RAG) explicitly conditions generation on retrieved evidence from a curated corpus (53). Often implemented as vector-based retrieval, graph-based RAG variants extend this idea by retrieving connected subgraphs that preserve relational context and reduce hallucinations when answering questions. For example, Graph-RAG frameworks have been used to generate requirements and specifications that are not only contextually relevant but align with complex higher level organizational or national requirements, and prior work shows they outperform baseline RAG methods (54). Mapping this to the 5C design process, knowledge-grounded operative systems act on structured representations (ontologies, knowledge graphs) to advance work products across multiple phases of design. In problem definition, retrieval-augmented and graph-based agents can actively surface missing, conflicting, or weakly supported requirements by traversing connected evidence (52). During conceptual design, such agents may propose candidate concepts, constraints, or reframings conditioned on retrieved precedents and design rationale (55). These systems do not merely represent information, they intervene in the design process by generating, checking, and updating artifacts and by orchestrating next actions based on retrieved, structured context.

Seen from a practice perspective, the representative–operative distinction is less of a hard boundary and more of a shift in where human initiative exists and how momentum is sustained. Representative tools help teams see more clearly (eg stitching evidence, quantifying uncertainty, and staging richer conversations) while operative systems keep the process moving by proposing, testing, and revising on their own cadence. Agency is always shared to some degree. For instance, humans might articulate aims, author objectives and constraints, and decide when to trust or override; agents could surface trade-offs we might miss and widen the search when our attention narrows. Recent ethnographic work by Ma et al. (56) demonstrates this balance, with qualitative evidence suggesting that professional designers adopt generative AI through distinct strategies, such as intimate codesign, selective delegation, and minimal use. This work highlights that generative AI and other operative technologies, results in a tempo that becomes continuous rather than episodic, and the locus of normativity moves from models alone to the objectives, rewards, and guardrails we design. This mixed-initiative rhythm echoes Simon’s view of design as *devising courses of action* (18).

From a design science perspective, operative technologies expose where value actually enters the system: in the articulation of objectives and constraints, in the balance of exploration versus exploitation, and in how search itself is structured. Just as in human teams, *how* an agent searches is as important as *what* it optimizes for; our recent work shows that steering cognitive style and exploration policy in LLM-driven design agents systematically changes the diversity and usefulness of solutions (57, 58). In this way, the adoption of operative technologies reframes the human engineer’s role from sole problem solver to a *steward of objectives and process*, responsible for curating data, constraints, and guardrails, and arbitrating trade-offs when machine-proposed actions conflict with broader social or organizational values (4).

#### Case study: operative technologies in consumer product innovation

Innovation in consumer products is often characterized by fast, early iterations and diffuse constraints (59), making this area a natural testbed for operative AI technologies. Rather than only improving the fidelity of evidence, operative systems increase the *bandwidth of available action*; they propose, test, and revise at a more rapid machine cadence while designers curate objectives, constraints, and guardrails at a reduced human cadence. In practice, teams progress from episodic concept reviews (often representative of low trust) to a more appropriately trusting mixed–initiative rhythm in which agents keep momentum and humans decide when to steer, stop, or escalate (57, 60, 61).

Here, we consider the alignment between the stages in the 5C design process and supporting representative operative AI technologies. In the “problem definition” stage, data-driven sensing and large-scale text analytics help surface user needs and perceptions at scale, for example, cyber-empathic pipelines that connect in-use signals to perceived attributes and ML-based mining of ecosystem reviews (62, 63). Representation tools translate noisy, unstructured feedback into structured design evidence by extracting usage contexts and estimating attribute importance–performance directly from reviews (64, 65). During “conceptual design,” knowledge representations (semantic networks, design knowledge graphs) act as computational “maps” that retrieve, organize, and relate prior solutions and concepts to catalyze human ideation without prescribing form (66, 67). In Embodiment Design, an intelligent system might run automated trade-offs and select embodiments for prototyping. And finally, in Value Proposition, human designers could adjudicate among agent-proposed solutions and choose those that align with brand, sustainability, or user-experience goals.

Beyond the academic studies noted above, a variety of industry case studies illustrate the impact of these technologies in practice. In consumer goods, L’Oréal, the world’s largest cosmetics manufacturer, has partnered with NVIDIA to use generative AI to rapidly develop and test product and marketing offerings, enabling designers and Research and Development teams to iterate on formulations, packaging, and brand experiences at unprecedented speed and significantly reduce time-to-market (68). Similarly, DeepMind’s reinforcement-learning-based system automates chip macro placement for tensor processing units (TPUs), producing layouts in hours that match or surpass expert human performance and have been deployed in production workflows (69, 70). These examples demonstrate how operative technologies are already generating, selecting, and orchestrating design decisions in high-stakes industrial contexts, with human designers increasingly focused on specifying objectives, curating alternatives, and validating machine-proposed solutions. At the same time, even in these fast-moving contexts, representative technologies are becoming essential for grounding such exploration. DT of user interaction, synthetic user models, and high-fidelity simulation environments provide feedback signals that enable agent-generated concepts to be evaluated prior to physical prototyping, refining both the solutions and the objectives that guide them. Together, these complementary roles form a tightly coupled exploration–validation loop, illustrating how integration of operative and representative technologies can accelerate innovation while maintaining alignment with real-world performance and user needs.

### Complementarity of technology types

Representative technologies increase the fidelity of design evidence, while operative technologies expand the actions available to designers by proposing, testing, and iterating at a rapid machine cadence. Used alone, the former can stall on data fidelity and interoperability, and the latter can drift through objective misspecification, compliance gaps, or provenance risks; used together, they create a closed-loop in which trustworthy models bound and instrument the search space as agents explore it under explicit guardrails and service-level objectives, as illustrated in Fig. 3. In this arrangement, humans remain central, arbitrating trade-offs when machine proposals intersect safety, sustainability, cost, or brand values. The practical payoff is earlier learning with fewer costly mistakes, representative tools derisk what to believe and operative tools derisk what to try. Critically, this distinction is not categorical but functional: the same method may serve a representative or operative role depending on how it is used. For example, function models, shape grammars, or generative systems may act as representations when they externalize structure, intent, or form for inspection and reasoning, or as operative mechanisms when they actively generate, transform, or select design alternatives within a workflow. Generative models that produce images, renderings, or CAD geometry may therefore be representative when they function as externalized design artifacts, and operative when embedded in closed-loop processes that drive exploration, evaluation, or downstream decisions. Closed-loop integration between these two technology types may begin first with representative models establishing a bounded, evidence-informed design space. Next, operative systems might generate and evaluate candidate solutions within those bounds, with outcomes validated against representative models and, where available, real-world or synthetic data. Finally, discrepancies between predicted and observed behavior could be used to update both the representative models and the objectives guiding operative search. This cycle could repeat across design phases, progressively tightening alignment between what is explored, what is known, and what is valued.

We highlight however, that although these technologies are complementary in principle, a variety of challenges still exist toward deeper integration of operative and representative technologies in practice. These can largely be divided into three categories: aligning representative technologies to the real world; aligning operative technologies to real value; and aligning operative and representative technologies with one another. First, representative technologies must align to a knowable degree with the real world, calibrated to physical behavior and operational context, or they risk creating persuasive but misleading evidence. Second, operative technologies must align with value: automated generation and selection must faithfully encode stakeholder priorities rather than optimizing narrow proxies. Third, operative and representative technologies must align with each other: agents should be bounded by what the representative models can credibly support, and representative models should be instrumented to provide the right feedback signals for agentic exploration and decision-making. In order to seamlessly integrate operative and representative technologies in practice, we must close all three of these gaps at the same time.

## What’s left for human designers?

Each successive industrial revolution has resulted in a paradigm shift in terms of humanity’s relationship with work and solutions to problems. The era of design for mass production changed the risk profile of products and hence, how designers approach creative problem solving. A single design decision (eg redesigning an existing aircraft versus a clean slate design), can have cascading effects on the final design solution. The question of what will be left for human designers is connected to whether designers will be designing for the masses or default back to designing for specific people or use cases. As AI and automation advance personalized learning, personalized medicine and other domains that adapt and optimize for a single user, will everyday products that designers currently design for the masses shift toward users designing and manufacturing for themselves with the support of AI agents and digital manufacturing technologies (ie 3D Printing) in the comfort of their homes, another twist on Schumacher’s “Small is Beautiful” production model (71)?

We can imagine a range of possible futures for how we design, based on why we design. Thinking of AI as automation, at one end of the automation spectrum is a case of hyper-automation, where machines overshoot humanity, efficiency is maximized, and human contribution and purpose are minimized. At the other end of this spectrum is some short of minimal automation, where we collectively decide to turn off many of our machines and return to a pastoral, if somewhat inefficient, existence. Both extremes are unlikely, but illustrate the tradeoff. Human values and desires will dictate the tradeoff on this automation spectrum, and designers will drive the implementation. A moderate-automation future may be most likely, in which designs make use of automation in appropriate ways. In perhaps the most hopeful future, we measure progress by how technology amplifies human well-being, creativity, and social cohesion, rather than by how much work can be automated. As in our past, design automation can relieve tedious, unsafe, and low-value tasks that constrain human potential.

From a design science perspective, this speculative spectrum defines the boundaries of designers’ responsibilities. Automation is not neutral, priorities and values are inherently embedded into automated systems (72), and design automation can ultimately constrain the agency of designers and redistribute creativity, judgment, and value. Herbert Simon anticipated that advances in computation and automation would not only reshape the means of design but also the moral and societal ends it serves. In his essay “Forecasting the Future or Shaping It” (73), he argued that as our capacity to design increasingly extends to the systems that govern life itself, the measure of progress must shift from technical achievement to ethical stewardship. He framed this responsibility through three conditions that define what he called a “pretty-well designed world”:

- Live at peace with all of nature.
- Share broadly and fairly the outputs of our labor.
- Mitigate and eliminate the divisions of “us against them.”

A designed future state must balance these objectives against pragmatic realities. The future of design work is no longer the creation of artifacts, but the orchestration of sociotechnical systems that increasingly design themselves. We view this shift through a guiding lens of closed-loop integration, grounded in three principles. First, bounded exploration, in which operative systems are constrained by the epistemic limits of representative models. Second, reciprocal updating, where exploration outcomes continuously refine those models and vice versa. Third, value alignment, where human designers calibrate objectives, constraints, and evaluation criteria to reflect stakeholder intent. Understanding the implications of this shift requires us to articulate what roles remain for designers across varying degrees of automation. The sections that follow explore four of these roles: human designers as stewards of intent, responsible for framing problems and values; as curators of exploration, guiding computational creativity and decision-making; as translators across systems, maintaining accountability and ensuring that the systems we design serve society’s broader goals; and as historians, archiving design processes and designerly knowledge.

### Human designers as stewards

Central to the design process is the discovery and synthesis of needs in the 5C problem formulation phase. This phase depends on deeply human capacities, such as the ability to understand others’ perspectives, negotiate conflicting values, and translate qualitative experiences into design features (the properties of the design solution that will be used by users and other stakeholders to judge if the solution actually solves the problem). The designer must map these features to actionable and measurable design objectives and requirements. Here, the designer serves as a steward of intent, guiding the design process with care and imagination, keeping the features central to decision making. The designer seeks to understand user needs while also anticipating how proposed solutions might affect broader social and environmental systems. Critically, designers must strike a delicate balance between empathy and reflective detachment, or more specifically, recognizing when meeting a single stakeholder’s needs might create new inequities or unintended consequences elsewhere. Representative technologies, such as DT, simulations, and immersive environments, can help designers navigate this balance by making complex systems visible and testable before physical realization. Operative technologies, such as elicitation and extraction of human preferences that are central tools in psychology and marketing have become design tools as well (74, 75). These tools extend human empathy and foresight, revealing interdependencies and potential consequences that designers might not otherwise realize. Yet, the realism or perceived fidelity of these technologies can be deceptive. They risk presenting partial models as complete truths, contributing to the propagation of potentially invalid or misaligned assumptions in the design process itself.

With the increased intelligence of computational agents, the human designer’s responsibility extends beyond shaping products to shaping the very data, objectives, and boundaries that inform machine behavior. We argue, that as design tools increasingly rely on data-driven models, human designers must ensure that the data used to train and guide these systems accurately reflect user needs and account for societal and ethical considerations, a critical area of focus within design science (76). The role of the designer-as-steward shifts from crafting artifacts to shepherding design intent, defining what problems should be solved, what trade-offs are acceptable, and whose voices are represented in the data. Without human stewardship, design risks becoming a process that optimizes for efficiency at the expense of empathy, equity, and care.

### Human designers as curators

If stewardship concerns the design intent, curation concerns design intelligence: how exploration unfolds once intent is established. Operative technologies, such as topology optimization and generative design, expand the design space to scales no human team could explore alone. With this power comes a new kind of cognitive responsibility: to decide what is worth exploring and what is worth discarding. In the mid-range of the future automation spectrum, we envision human designers will act as curators of exploration, setting objectives, constraints, and stopping conditions that guide machine search processes. Design creativity within human-AI teams is a distributed process that depends on interaction between divergent and convergent reasoning (32, 77) between human intuition and machine computation. We hypothesize that the designer’s role will shift from solving problems directly to framing them meaningfully and interpreting the results of automated search with judgment and context. This shift may impact the number of designers needed to perform such tasks or the level of expertise of designers needed to serve these evolving roles.

### Humans as translators

While curation emphasizes how humans frame and interpret within computational design processes, mediation extends this role outward, connecting not only humans and machines, but the social, ethical, and organizational systems in which design unfolds. Designers must translate between languages of intent and execution, between human-centered values and computational logic, and between the abstractions of models and the lived realities of users. In this context, translation is the act of preserving meaning and intent as information moves across representational and operative layers of design.

Representative technologies translate physical systems into digital form, while operative technologies translate objectives into action. Between these layers lies a crucial human task: ensuring that the meaning embedded in these translations remains aligned with human needs, ethical principles, and social contexts. Without this interpretive layer, the design process risks becoming a closed feedback loop in which optimization displaces understanding. Designers working as translators, between computational agents and other systems, ensure that computational efficiency does not erase the nuances of human experience. Further, designers increasingly work at interfaces where engineering models, policy frameworks, and user practices intersect. In these contexts, the designer’s role is to reconcile incompatible vocabularies, building, as Bucciarelli noted (19), *boundary objects* that help reconcile the precision of technical models with the ambiguity of human judgment across domains. Research in systems design underscores that such translation is essential for avoiding fragmentation in large-scale systems and for maintaining shared situational awareness across subgroups (78).

We draw a parallel here to the concept of tolerance stack-up from geometric dimensioning and tolerancing (GD&T). In GD&T, a tolerance stack-up describes how small, acceptable variations in individual parts can accumulate across an assembly, ultimately resulting in a system that is out of tolerance, even when each component, taken alone, conforms to specifications. A similar phenomenon can emerge in computationally driven design and has already been cited as a concern in DT design environments (23). As user needs, design concepts, and engineering requirements are translated into machine-readable data and then back into human-interpretable outputs, or vice-versa, each translation step may appear “within tolerance.” In aggregate, however, these translations can drift, distorting meaning until the final design no longer aligns with its original intent.

Here, the criticality of the *designer-as-translator* becomes evident. Designers are not merely intermediaries converting between representational forms but are responsible for monitoring how meaning and value shift across the design process. In this midautomation future, the designers’ role is to detect and correct these cumulative drifts and ensure that, despite layers of automation and abstraction, the final outcome remains “within tolerance” with respect to human values, social responsibility, and design intent.

### Everyone as designers and designers as historians

As we explore possible futures of design science and the role of designers, one must consider the possibility that designers would no longer be necessary as a profession or discipline in the age of AI and automation, as these technologies may result in the *democratization of design*. In other words, as these technologies become more commonly adopted into the general life and workflow of the human population, humans will inevitably use these tools to problem solve, changing their current situations into more desirable or preferred situations, and from the perspective of Simon, becoming a designer. If everyone is a designer, then design as a profession may cease to exist. If the reason why specific professions exist is to solve a human need, then the obsolescence of a profession or discipline could serve as evidence of a need being solved. We argue, that to hold onto a discipline simply because one has been trained in it could stifle human creativity and societal progress. There may very well be a future where the very small subset of the human designers that remain, serve as historians that share knowledge about the origins and evolution of human designers, rather than design solutions to user needs.

Take, for example, the popularity of desktop 3D printers and the proliferation of online CAD tools, which in combination have enabled anyone, even children (79), to design and manufacture their own products. Integrating advanced representative and operative technologies into this current state, it is easy to envision a future in which an individual simply converses with an AI agent: describing a need, receiving concept sketches and simulations in real time, refining requirements through natural language, and ultimately producing a ready-to-fabricate model generated through an agent-led optimization. In such a world, design expertise becomes ambient rather than professionalized. The specialized knowledge once held by trained designers is partially absorbed into ubiquitous tools, leaving only a small sample of experts who contextualize, interpret, and preserve the history and evolution of design practice itself.

Rather than creating solutions, these remaining designers may serve as historians who document how human intention, creativity, and cultural values once shaped the built world, and how those practices transformed as machines assumed greater responsibility in defining and realizing preferred states. This shift does not diminish the importance of design but reframes it as a shared human capacity, augmented by computation.

## Advancing design science in the age of automation

Design science becomes essential not only as a means to improve processes but as a framework for understanding the consequences of automation. It offers the theoretical and methodological tools to study how human purpose, creativity, and responsibility can be embedded within computational systems without being erased by them. The utility of the human designer may no longer lie in producing solutions faster or cheaper, but in ensuring that design outcomes remain aligned with human values. Future research must therefore move beyond the question of how to make AI better *at* design and instead ask how to make design better *with* AI. This requires reframing design theory to account for hybrid intelligence, or systems in which humans and machines jointly frame problems, generate alternatives, and negotiate trade-offs. It also demands new methodological approaches to study the permutations of values and intent through a computationally augmented or driven design process. For example, future work might explore how biases propagate through design algorithms, derive frameworks for auditing automated design decisions, or investigate participatory methods that ensure diverse stakeholder values are represented in computational objectives (76).

As automation reshapes the nature of design work, research and practice must address its human dimension: the dignity, agency, and meaning of design labor in a cocreative world. For practitioners, this means re-examining what constitutes meaningful contribution when creative and analytical tasks are shared with machines. It requires cultivating practices that preserve reflection, empathy, and human judgment as essential complements to computational efficiency. Designers will need to shift perspective, from viewing emerging technologies as mere tools to recognizing them as active participants in the design process. Just as designers are attuned to the prior experiences and perspectives that human collaborators bring to a team, often requesting teammates based on these qualifications, they must also critically evaluate the values, assumptions, and biases embedded within both representative and operative technologies. Operative technologies, in particular, challenge designers to engage with what machines produce but also with how they produce it, specifically the objectives they optimize, the trade-offs they encode, and the boundaries they impose on creativity. Collaboration with intelligent systems, therefore, demands a reimagination of design education. Preparing the next generation of designers will require more than technical fluency; it will require cultivating ethical judgment, reflection, and interpretive skill. Design curricula must teach students not only how to work with intelligent systems, but how to question, critique, and redirect them. These efforts will be critical in developing designers who do not blindly trust automation but can effectively criticize automation and intervene when appropriate or necessary. Thus, we believe the future of engineering design education must work hand-in-hand with the humanities to integrate critical thinking curricula.

For researchers, this calls for frameworks that capture the experiential and ethical dimensions of design work in automated contexts, investigating how human motivation, authorship, and identity evolve as machine participation increases. It demands novel research methods that enable data collection and empirical studies of how designers interact with, shape, and are shaped by automated systems, working to link microlevel design behaviors to macrolevel social, ethical, and organizational outcomes. For example, critical to the successful integration of these technologies in design is a more thorough understanding of the fundamental ways in which human designers form and maintain trust in technology driven counterparts, such as AI agents or DT. Researchers within design science should actively collaborate with peers in Human–Computer Interaction, Cognitive Psychology, and Artificial Intelligence to develop empirically grounded models of trust that account for both the capabilities and limitations of opaque learning systems. Rather than assuming that trust emerges from transparency alone, future work must examine how designers calibrate trust through experience, performance history, uncertainty communication, and the ability to interrogate assumptions, provenance, and failure modes.

We argue that representative and operative technologies provide rich contexts for development of novel frameworks, methods, and theories to capture design work in automated context. DT and immersive simulations offer new ways to observe and model human–machine collaboration, while generative and agentic systems create opportunities to study how computational agency transforms creativity, decision-making, and the design practitioners’ responsibilities when embedding artifacts in our lives. Further, the old demands for collaboration among design research, education, and practice, as well as among industry, government, and community, are much more urgent now, as automation increases the speed of change and reduces the time margins for recovery from unforeseen consequences.

## Conclusion

The contribution of this perspective is not the introduction of a new method, algorithm, or process, but a reframing of how emerging AI capabilities collectively reshape design practice and design science. By distinguishing between representative and operative technologies and situating both within the 5C model, we provide a unifying framework that clarifies where technology intervenes in design processes, how it alters epistemic risk and exploration dynamics, and why human designers remain essential as stewards of intent, value, and accountability. This framing exposes new research questions concerning trust, responsibility, and the redistribution of agency in increasingly automated design systems. In doing so, this Perspective offers design science a conceptual scaffold for studying advanced technologies in design, and for guiding future empirical, methodological, and educational efforts as design evolves toward hybrid human–machine systems.

Design increasingly occurs in hybrid human–machine teams. Representative and operative technologies expand what can be known, generated, and evaluated, while also redistributing agency and responsibility. The path forward is to embrace automation while retaining human stewardship over intent, values, and accountability throughout the design process.

## Data Availability

There is no data underlying this work.
